# Functional Genomics of Healthy and Pathological Fetal Membranes

**DOI:** 10.3389/fphys.2020.00687

**Published:** 2020-06-19

**Authors:** Sarah J. Cunningham, Liping Feng, Terrence K. Allen, Timothy E. Reddy

**Affiliations:** ^1^Department of Biostatistics and Bioinformatics, Duke University, Durham, NC, United States; ^2^University Program in Genetics and Genomics, Duke University, Durham, NC, United States; ^3^Center for Genomic and Computational Biology, Duke University, Durham, NC, United States; ^4^Center for Advanced Genomic Technologies, Duke University, Durham, NC, United States; ^5^Department of Obstetrics and Gynecology, Duke University School of Medicine, Durham, NC, United States; ^6^Department of Anesthesiology, Duke University Hospital, Durham, NC, United States

**Keywords:** genomics, fetal membranes, transcriptomics, preterm birth, gene regulation and expression

## Abstract

Premature preterm rupture of membranes (PPROM), rupture of fetal membranes before 37 weeks of gestation, is the leading identifiable cause of spontaneous preterm births. Often there is no obvious cause that is identified in a patient who presents with PPROM. Identifying the upstream molecular events that lead to fetal membrane weakening presents potentially actionable mechanisms which could lead to the identification of at-risk patients and to the development of new therapeutic interventions. Functional genomic studies have transformed understanding of the role of gene regulation in diverse cells and tissues involved health and disease. Here, we review the results of those studies in the context of fetal membranes. We will highlight relevant results from major coordinated functional genomics efforts and from targeted studies focused on individual cell or tissue models. Studies comparing gene expression and DNA methylation between healthy and pathological fetal membranes have found differential regulation between labor and quiescent tissue as well as in preterm births, preeclampsia, and recurrent pregnancy loss. Whole genome and exome sequencing studies have identified common and rare fetal variants associated with preterm births. However, few fetal membrane tissue studies have modeled the response to stimuli relevant to pregnancy. Fetal membranes are readily adaptable to cell culture and relevant cellular phenotypes are readily observable. For these reasons, this is now an unrealized opportunity for genomic studies isolating the effect of cell signaling cascades and mapping the fetal membrane responses that lead to PPROM and other pregnancy complications.

## Introduction

Preterm birth remains a major public health challenge affecting 10% of pregnancies in the United States (World Health Organization, 2016). The leading identifiable cause of preterm birth is premature preterm rupture of membranes (PPROM) (Mercer, 2010). Preeclampsia is characterized as shallow trophoblast invasion leading to incomplete spiral artery remodeling. It affects 5% of pregnancies and is an iatrogenic cause of prematurity and the leading cause of maternal and perinatal death (Souza et al., 2013). These adverse pregnancy outcomes all have multifactorial causes incorporating genetic and environmental risk factors.

Functional genomics assays aim to define the relationships between the human genome and epigenome; the environment; and molecular, cellular, and organismal phenotypes. The past decade has been transformative for functional genomics, owing largely to high-throughput short read sequencing providing quantitative and genome-wide readout for many functional genomic assays. Such assays are particularly adept at identifying differential activity that may result from changes in the environment, such as hormone exposures or immune insults [e.g., (McDowell et al., 2018; Pulido-Salgado et al., 2018)]. Today, there are a vast array of genome-wide functional genomic technologies available to measure a wide variety of aspects of gene expression, DNA methylation, histone positioning and modifications, transcription factor binding, gene regulatory activity, other factors that indicate gene regulation (Arnold et al., 2013; Mundade et al., 2014; Finotello and Di Camillo, 2015; Tirado-Magallanes et al., 2016). Through those studies, there is now extensive information about the gene regulatory state of diverse cells and tissues.

For the purposes of this review, we define functional genomic assays as those that scan large fractions of the genome for evidence of regulatory activity. In the context of human disease studies, such regions are a promising starting point for subsequent efforts to discover causative biological mechanisms. Follow-up is then needed to evaluate the biological consequences of identified regulatory regions, both in terms of the effects on cellular and organismal phenotypes and also in terms of the effects of non-coding genetic variation on their activity.

Functional genomics studies have primarily focused on immortalized cell models, ostensibly because they are highly proliferative and robust. However, recent advances in the adaptation of functional genomics protocols for use on limited primary cells and tissues have created the potential to study more physiologically relevant cell models [e.g., (Vento-Tormo et al., 2018; Chung et al., 2019)]. In the context of preterm birth, fetal membranes are a key tissue of interest, and ideally suited for genomic analysis due to their availability and amenability to cell culture. Protocols to culture and expand amnion and chorion cells were developed in the 1980s (Burgos and Faulk, 1981). Culturing primary cells in these systems allows for interrogation of fetal membranes by genomic assays. In addition, several genomic assays are now feasible from a limited number of primary cells, and even single cells, making culturing unnecessary (Jia et al., 2018; Wang et al., 2019). Together these developments have led to a number of genomic assays comparing fetal membrane tissues from healthy pregnancies to those involved in preeclampsia, early pregnancy loss and preterm birth.

## Defining Fetal Membrane Specific Regulatory State

Functional genomic assays on fetal membrane samples have been completed both by large genomics consortia such as ENCODE and Roadmap Epigenomics (The Encode Project Consortium, 2012; Roadmap Epigenomics Consortium et al., 2015), as well as by individual labs (Kim et al., 2012; Lim et al., 2012; Table 1). The consortia efforts have focused on amnion and chorion tissues from full term and second trimester samples. Across those samples, they have measured genome-wide gene expression using RNA-seq, cytosine methylation using whole genome bisulfite sequencing, and the locations of covalent histone modifications, indicators of active gene regulation, using ChIP-seq.

**TABLE 1:** Published genomic analyses in fetal membrane tissues.

**Source**	**Tissue Type**	**Disease State**	**Assay**
Roadmap Epigenomics	Amnion, basal plate, chorion smooth, trophoblast, placental villi	Healthy full term and 2nd trimester c-sections	mRNA-seq, histone modification ChIP-seq
ENCODE	Amnion, basal plate, chorion, trophoblast, placental villi	Healthy full term and 2nd trimester c-sections	mRNA-seq, microRNA-seq, DNase-seq, histone modification ChIP-seq
Tromp et al., 2004	Chorioamnion	Preterm Labor, PPROM, Term in labor and term not in labor	Microarray
Montenegro et al., 2009	Chorioamnion	Term in labor and not laboring and preterm labor	microRNA microarray
Nhan-Chang et al., 2010	Amnion and Chorion	Healthy full term spontaneous rupture of membranes	Microarray
Li et al., 2011	Amnion mesenchymal cells	Healthy term c-section stimulated with IL-1B	Microarray
Kim et al., 2012	Amnion, chorion	Healthy full term c-section	RNA-seq
Kim et al., 2013	Amnion	Term in labor and not laboring and preterm labor	Illumina Methylation BeadChip
Lim et al., 2012	Amnion	Healthy full term c-section	Microarray
Kim et al., 2016	Amnion epithelial	Healthy and preeclamptic term c-section	mRNA-seq
Sõber et al., 2016	Chorionic villi	Recurrent pregnancy loss and elective abortion 2nd trimester	mRNA-seq, MicoRNA-seq
Wang et al., 2017	Chorionic villi	Recurrent miscarriages and elective abortion	lncRNA microarray
Jiang et al., 2018	Chorionic trophoblasts	Healthy term c-section stimulated with LPS	RNA-seq, Whole Genome Bisulfite Sequencing
Pereyra et al., 2019	Amnion and Chorion	Severe preterm and full term spontaneous labor	RNA-seq
Yang et al., 2019	Chorionic villi	Early embryonic arrest and elective abortion 2nd trimester	mRNA-seq, MicoRNA-seq

RNA-seq typically measures mRNA transcript levels that can be compared among tissue types to identify tissue specific transcription (Mortazavi et al., 2008). Bisulfite sequencing assays identify methylated cytosine that are typically thought to be related to silencing of gene activation. They do so by using sodium bisulfite treatment to convert unmethylated cytosines to uracil prior to PCR amplification and sequencing (Meissner et al., 2008). When sequencing treated and untreated DNA, the uracil bases sequence as thymine in treated samples but remain as cytosine in untreated samples (Clark et al., 1994). Finally, ChIP-seq assays use antibodies to isolate DNA-bound proteins including histones (Johnson et al., 2007; Robertson et al., 2007). ChIP-seq can detect histone subunits altered with post translational modifications that influence DNA affinity and, in turn, how accessible the DNA is to transcriptional machinery (Zhou et al., 2011). Together, these datasets can establish a baseline of gene regulatory state across among membranes from healthy pregnancies, and an assessment of the changes in gene regulation between the second and third trimester.

Additional studies have investigated the gene expression (Kim et al., 2012) of healthy term placental tissue types including fetal membranes. Transcriptomic analysis shows that placental cell types are more similar to each other when compared to other adult tissue types, but each placental cell type shows a subset of tissue type specific gene expression as well (Kim et al., 2012). The epithelial specific splice regulator ESPR1 is significantly unregulated in fetal membrane tissue, particularly the amnion, above other tissue types. In the amnion, the relative expression of ESPR1 is 50% higher than that of next highest tissue of the 16 adult tissues measured. Substantial alternative splicing and novel isoforms specific to the fetal membranes have been found by RNA-seq studied of healthy term membranes (Kim et al., 2012).

To further define healthy gene regulation in fetal membranes, microarray-based gene expression studies compared activated amnion from late term non-laboring elective Cesarean-sections, defining activation as high NF-κB protein levels similar to the levels observed in post-delivery samples (Lim et al., 2012). That activation of the amnion is an early step that stimulates the synthesis of prostaglandins, cytokines and chemokines initiating the beginning of labor. Although all the samples were non-laboring, some samples were closer to the onset of labor at the time of C-section and could be differentiated from more quiescent samples. Activation of the amnion is associated with an up regulation of a cell death and cancer associated gene network, consistent with an increase in apoptosis in activated amnion (Lim et al., 2012). An additional gene network associated with cell-to-cell signaling is also unregulated in response to activation, consistent with the role of the amnion as an early initiator of labor induction.

## Healthy Versus Pathological Fetal Membranes

Functional genomic studies are particularly powerful for identifying molecular differences between different cell states, such as between fetal membrane tissues from healthy pregnancies and those with pregnancy complications. Differentially regulated genes from these studies can identify molecular pathways that may be either causal or a downstream consequence, and in the best cases can nominate new therapeutic targets. In fetal membrane tissues, such comparative studies have been done to compare healthy tissues to those from patients with preterm birth, recurrent early pregnancy loss, and preeclampsia.

### Preterm Birth and Membrane Rupture

Due to the integral role fetal membranes play in maintaining pregnancy or stimulating parturition, a substantial amount of research has been devoted to understanding changes in gene regulation that occur during the onset of parturition. RNA-seq analyses have revealed hundreds of gene expression changes that occur between the site of membrane rupture and distal membrane sites in term spontaneously ruptured membranes. For example, Nhan-Chang et al. (2010) used microarrays to identify 677 differentially expressed genes at the site of rupture compared to a distal site in the chorion (Nhan-Chang et al., 2010). The differentially expressed genes were enriched for increased expression of genes involved in complement and coagulation at the site of rupture, suggesting a role for immune activation in membrane integrity. Genes related to extracellular matrix-receptor interaction were most altered at the site of rupture, consistent with the role of the extracellular matrix in maintaining fetal membrane integrity (Bryant-Greenwood, 1998).

Because signaling cascades leading to fetal membrane rupture can be informative in identifying the causes of membrane rupture, directly comparing the gene expressed at the site of rupture in preterm and term deliveries can give more direct insight into the cause of PPROM. The gene expression patterns of term membrane samples are more internally consistent whereas preterm samples are more variable (Pereyra et al., 2019). The variability in preterm samles suggests multiple signalling cascades lead to preterm birth, distinct from those leading to term births. Despite this variability, 270 significantly differentially expressed genes with a >2-fold gene expression change were found when comparing membranes from early preterm births to membranes from term births. Several genes from the tumor necrosis factor (TNF), chemokine and voltage gated potassium channel families were significantly differentially regulated. Inflammatory and immunological pathways were also significantly up-regulated in preterm birth, consistent with a role for immune responses in the etiology of some preterm birth (Velez et al., 2008).

Functional genomic studies can also identify genes of interest for specific causes of preterm birth. In one example, comparisons of gene expression in fetal membranes between preterm labor with intact membranes and membranes from PPROM patients identified Proteinase Inhibitor 3 (PI3) having significantly decreased expression in PPROM samples (Tromp et al., 2004). Immunohistochemical staining confirmed the decreased expression levels of PI3 protein expression in fetal membranes collected from patients presenting with PPROM (Tromp et al., 2004). PI3 is an anti-proteinase that may protect the extracellular matrix from degradation by proteases, specifically Elastase 2 and Proteinase 3 (Guyot et al., 2005). In other cell types, TNFα and IL1β have been found to induce PI3 production (Pfundt et al., 2000; Bingle et al., 2001). PI3 was not previously implicated in preterm birth or PPROM specifically, but genome wide studies suggest decreased expression, due to genetic or environmental signaling, could lead to PPROM.

Functional genomic studies have also identified differential epigenetic states which may contribute to or result from such gene expression differences. Differential DNA methylation in amnion between term and preterm pregnancies in labor and term pregnancies not in labor show the majority of changes in methylation occur at the onset of labor. A large portion of the differentially methylated genes are associated with non-coding RNA and imprinted genes (Kim et al., 2013). Of the regions that show changes in methylation between preterm and term, enrichment in genes related to cation transport, cytokine production and extracellular matrix receptor interactions were observed, supporting differential expression studies demonstrating similar patterns in functional enrichment (Kim et al., 2013).

MicroRNAs, another layer of control, regulate gene expression post-transcriptionally through binding to and destabilizing mRNA molecules (Ambros, 2004). Although most miRNA lack experimentally validated targets, computational predictions can suggest genes that may be involved in biological processes (Ekimler and Sahin, 2014). Ten miRNA were specifically differentially regulated between term in labor and preterm labor membranes, all of which were down regulated (Montenegro et al., 2009). Additionally, the RNA processor Dicer was down regulated suggesting miRNAs play a key role in the parturition process at term but not preterm. Coupled with gene expression data, these studies can show regulation that occurs at the onset of labor that separates preterm and term processes.

### Early Pregnancy Loss

Early embryonic arrest affects approximately 10% of pregnancies with rates increasing as the age of couples trying to conceive increases (Larsen et al., 2013). Pregnancy loss is due to factors including uterine abnormalities, abnormal chromosomes and infection pathologies but genetic factors can also lead to a pregnancy loss (Xu et al., 2016). Transcriptomic profiles from chorionic villi of early embryonic arrest samples compared to gestation age matched elective termination samples show differentially expression in PI3K-Akt signaling pathway, Jak-STAT pathway and complement and coagulation signaling cascades (Yang et al., 2019). One study looking specifically at long non-coding RNA that are differentially regulated in chorionic villi between patients with recurrent miscarriage and those undergoing an elective abortion found up regulation of steroid hormone biosynthesis and extracellular matrix interaction and well as down regulation of TGF-beta signaling and apoptosis pathways (Wang et al., 2017). An additional study comparing chorionic villi from recurrent pregnancy loss couples, defined as having five or more miscarriages, to elective termination samples shows a substantial down regulation of key small non-coding RNA as well as histone genes (Sõber et al., 2016). Those results suggest that chorionic villi cells begin repressing key cellular processes leading to loss of the pregnancy.

### Preeclampsia

Preeclampsia is a common disease that is the leading cause of pregnancy associated mortality and morbidity for both the mother and child (Roberts and Cooper, 2001). Shallow trophoblast invasion and impaired remodeling of the uterine spiral arteries are associated with preeclampsia (Pennington et al., 2012). Gene expression of amnion epithelial cells from healthy and preeclamptic c-sections were compared to understand the underlying disease etiology. Functional annotation of differentially expressed genes identified pathways involved in extracellular matrix-receptor interaction and focal adhesion. Additional validation studies showed differential expression of matrix metalloproteinases that control degradation of the extracellular matrix (Kim et al., 2016).

## Response to Stimuli

Understanding signaling events that cause membrane rupture can suggest specific pathways misregulated in PPROM. Testing specific response pathways can connect early signaling events from *in vitro* stimulus response studies to *in vivo* studies that examine the progression of labor. Such *in vitro* studies can circumvent the limitation that observational studies are necessarily correlative and thus cannot differentiate between the cause and consequence. Additionally, *in vitro* stimulus-response studies can identify intermediate steps leading to the onset of phenotype that observational studies miss due to strict limits on tissue collection during pregnancy. For example, *in vitro* functional genomic studies of fetal membranes cells responding to inflammatory stimuli can reveal the direct effects of those signals on pathways related to cell proliferation, adhesion, or apoptosis that may impact the timing of membrane rupture. Indeed, studies of cultured amnion mesenchyme cells exposed to an IL1β challenge for up to 8 h showed transcriptional dynamics reflecting an immediate immune challenge compared to sustained response. The early responsive genes showed signatures of NF-κB activity, a well-documented effector of IL-1 signaling (Cogswell et al., 1994; Greten et al., 2007; Liu et al., 2017). Later responsive genes had more diverse transcription factor binding sites indicative of a cascade of downstream gene regulatory events. Those secondary factors including the AP-1 family transcription factors that were not regulated by the initial IL-1β response (Li et al., 2011). Similarly, immune challenges to chorionic trophoblast cells through lipopolysaccharide (LPS) show an increase in gene expression related to cytokine production and response, although this signaling appears to be mediated through the STAT1-STAT3 pathway (Jiang et al., 2018). While differential DNA methylation is detected following LPS stimulation, 2 h of LPS induction may not be enough to detect significant changes in methylation. Together, these studies demonstrate the types of insights possible from functional genomic studies of fetal membrane cells after *in vitro* exposures. However, many of the common signals in pregnancy such as hormonal changes, oxidative stress and mechanical force changes remain to be investigated.

## Genetic Studies of Fetal Membranes

Transcriptomic and DNA methylation studies can take on additional informative power when combined with genetic association studies. Most variants identified in genome wide association studies are found in non-coding regions (Zhang and Lupski, 2015). Integration with functional genomic data sets can reveal candidate causal mechanisms, including target genes of clinical importance (Lowe and Reddy, 2015). The primary challenge is that the lead signal in a genetic association study is in linkage disequilibrium with many surrounding variants. Thus, the patterns of linkage disequilibrium in the study population limit resolution, often to >10 kb. Functional genomic datasets can suggest which variants in that LD-based region are most likely to have regulatory activity (Conde et al., 2013). That approach was used to identify a variant that abolishes a transcription factor binding site that represses interleukin 1 family members in fetal membranes (Liu et al., 2019). The variant identified was suggested to have a gene expression effect on multiple members of the interleukin 1 family including IL1A, IL36G, and IL36RN. A similar approach was also used in a genome wide association study of early preterm and term infants. Several significant variants near the gene SLIT2 were identified that overlaps regions of DNase hypersensitivity, suggesting regulatory activity, in several fetal tissues including the amnion (Tiensuu et al., 2019).

The combination of epigenomic data and genome wide association studies has also been employed for other pregnancy complications affecting the fetal membranes, including preeclampsia. A genome wide association study that incorporated both maternal and fetal DNA variants identified a variant near the gene FLT1 from the offspring of pregnancies associated with preeclampsia (McGinnis et al., 2017). The evidence for the effect of this variant was built by the fact that Roadmap Epigenomics incorporating many different epigenetic datasets, such as histone modifications and open chromatin sites, labeled this site as a putative enhancer in both amnion and trophoblast cell types.

While many genome wide association studies detect common non-coding variants from large populations, rare coding variants can also contribute to disease. In these cases, whole exome sequencing is often employed to detect these variants. A whole exome sequencing study of PPROM cases and healthy term controls in an African American population identified 10 rare variants more common in PPROM cases than term controls in native regulators of innate immunity, LPS detoxifying enzymes and antimicrobial protein genes (Modi et al., 2017). An additional follow up replication study replicated two of the variants in the genes DEFB1 and MBL2, both thought to be antimicrobial proteins in fetal membranes (Modi et al., 2018). The use of genomic sequencing technologies can detect both common and rare variants associated with fetal membranes pathologies. Studies identifying variants relevant to these pathologies are outlined in Table 2.

**TABLE 2:** Published genetic analyses of preterm birth.

**Source**	**Disease State**	**Assay**
McGinnis et al., 2017	Offspring of preeclamptic pregnancies and controls	SNP array
Modi et al., 2017	Healthy term and PPROM African American infants	Whole Exome sequencing
Modi et al., 2018	Healthy term and PPROM African American infants	Whole Exome sequencing
Liu et al., 2019	Varied gestation duration	SNP array
Tiensuu et al., 2019	Term and spontaneous preterm birth	SNP array

## Future Studies

While the number of studies comparing regulation between healthy and pathological membranes is growing, the data available remains sparse. Published studies have largely focused on comparing transcriptomic data or DNA methylation between cases and controls, often using microarray measurements that are noisier and have less dynamic range than sequencing-based methods (Zhao et al., 2014). In addition, few studies on fetal membranes have deposited raw data in publicly available databases, limiting benefit to other fetal membrane researchers. All together missing are assays of chromatin accessibility or histone modification in fetal membrane tissue type which can add more information about different levels of regulation and suggest transcription factors responsible for signaling that leads to pregnancy complications. Expanding studies of the response to relevant stimuli in fetal membrane tissues is a major opportunity. Studies thus far have focused on cellular responses to inflammatory stimuli but further studies looking at mechanical stress, hormone signaling and oxidative stress using *in vitro* tissue models in addition to cellular models to replicate the structural complexity of fetal membranes and cellular interaction can help add to a more complete understanding of the signaling that leads to PPROM, preterm birth, preeclampsia or early pregnancy loss.

## Author Contributions

SC wrote the manuscript with supervision from TR. TA and LF edited the manuscript. All authors contributed to the article and approved the submitted version.

## Conflict of Interest

The authors declare that the research was conducted in the absence of any commercial or financial relationships that could be construed as a potential conflict of interest.
